# A rare complication of hemolacria after Le fort I osteotomy: a case presentation

**DOI:** 10.1186/s40902-022-00359-1

**Published:** 2022-09-17

**Authors:** May S. Helal, Ramy M. Gaber, Marwa El-Kassaby

**Affiliations:** 1 Oral and Maxillofacial Surgery, Faculty of Dentistry, Alexandria University, Alexandria, 21531 Egypt; 2 Oral and Maxillofacial Surgery, Faculty of Dentistry, Ain Shams University, Cairo, Egypt

**Keywords:** Hemolacria, Bloody tears, Retrograde hemorrhage, Nasolacrimal, Le fort I

## Abstract

**Background:**

Nasolacrimal duct obstruction (NDO) is a common pathology preventing the proper drainage of the tears, and its main symptom is epiphora. Secondary acquired nasolacrimal duct obstruction (SANDO) can be due to a variety of causes including infection, trauma, or neoplasms. It has been reported to occur with different forms of maxillofacial trauma, especially Le Fort II, Le Fort III, naso-orbital-ethmoidal, and orbital floor fractures.

**Case presentation:**

A 20-year-old Egyptian female presented to correct a facial disharmony due to a cleft lip and palate defect. The patient reported a history of congenital NDO and had deficient lateral nasal walls. Bimaxillary surgery was planned, including a Le Fort I osteotomy for the maxilla and bilateral sagittal split osteotomy for the mandible. The surgery was uneventful, but the patient complained from bloody tears or hemolacria few days postoperatively. This complication began to cease spontaneously after 2 days and completely recovered after 4 days.

**Conclusion:**

Hemolacria is an infrequent finding after maxillofacial surgeries and may be associated with CLP surgeries more than other surgeries. In this case, it was easily managed, and surgeons should be more aware of it to try to prevent its occurrence.

## Background

The lacrimal apparatus is composed of the lacrimal glands to produce the tears, the lacrimal sac, and the nasolacrimal duct for drainage of the tears into the inferior meatus of the nose [1, 2]. The medial border of the nasolacrimal duct is the palatine bone and inferior nasal turbinate, and the lateral border is the maxillary bone. The embryological origin of the duct is an ectodermal thickening found between the maxillary and lateral nasal prominences [3].

Nasolacrimal duct obstruction (NDO) is a common pathology preventing the proper drainage of the tears. The main symptom of NDO is epiphora, meaning an overflow of tears onto the face without any trigger or due to pressure or “continuous accumulation of tears in the lacrimal lake, due to an impeded outflow” [4–6]. It can happen congenitally, when canalization fails to occur in embryologic development. This is more commonly associated with other congenital anomalies such as Down syndrome, cleft lip and palate, hemifacial microsomia, and Treacher Collins syndrome [3, 7, 8].

In adults, NDO can either be a primary entity, with no apparent cause, or it can be a secondary NDO [9]. The etiology of primary acquired nasolacrimal duct obstruction (PANDO) is unknown but may be attributed to a narrow bony canal for the nasolacrimal duct pathway [10]. Secondary acquired nasolacrimal duct obstruction (SANDO) can be due to a variety of causes including infection, trauma, or neoplasms [5, 6]. It has been reported to occur with different forms of maxillofacial trauma, especially Le Fort II, Le Fort III, naso-orbital-ethmoidal, and orbital floor fractures [6].

The purpose of this study is to present a case of hemolacria occurring after Le Fort I (LF-1) osteotomy was performed, for the correction of maxillomandibular disharmony in a cleft lip and palate (CLP) patient. To the authors’ knowledge and to this date, this complication was only reported in four other cases throughout the literature [4, 11, 12].

## Case presentation

A 20-year-old Egyptian female presented to the Cleft Center, Ain Shams University, seeking for better facial esthetics. She had a history of various operations for amendments of a cleft lip and a cleft palate defect, at various points of her growth timeline, which may indicate the presence of a congenital NDO and which may be due to (or not) the presence of the cleft deformity. The patient’s mother reported previous multiple occurrences of epiphora during the patient’s childhood, leading to an ocular surgery being performed to treat this symptom.

The patient presented with a remaining anterior palatal fistula, and an angle class III maxillomandibular, causing a concave profile appearance of the face. Facial features included widened and flattened nasal bridge. The treatment option was a surgical decision to perform bimaxillary surgery, including a LF-1 osteotomy in the maxilla, and bilateral sagittal split osteotomy (BSSO) in the mandible. The plan was to move the maxilla forward and the mandible backwards to correct the maxillomandibular relation.

The required investigations and radiographic and clinical examinations were collected before the surgery, including a cone-beam computed topographical (CBCT) scan. It is an important notice that the CBCT scan revealed deficient lateral nasal walls, which was attributed to the CLP defect, and proper consents taken from the Institutional Review Board of the Ain Shams University.

The patient was admitted to the operations room, and general anesthesia was induced in patient by nasotracheal intubation. Maxillary vestibular incision and the LF-1 osteotomy were carried out. LF-1 osteotomy started from the lateral nasal rim, 3–4 mm above the nasal floor, and extended to the pterygomaxillary junction, 5 mm above the teeth roots. The pterygoid plates were separated, and osteotomies were completed in the lateral nasal walls, followed by downfracture. The incision and osteotomies were completed without any obvious complications or deterrents. Incision along the anterior border of the ramus and external oblique ridge was executed to allow the BSSO osteotomy. All incisions were sutured properly at the end of the surgical operation.

**Fig. 1:**
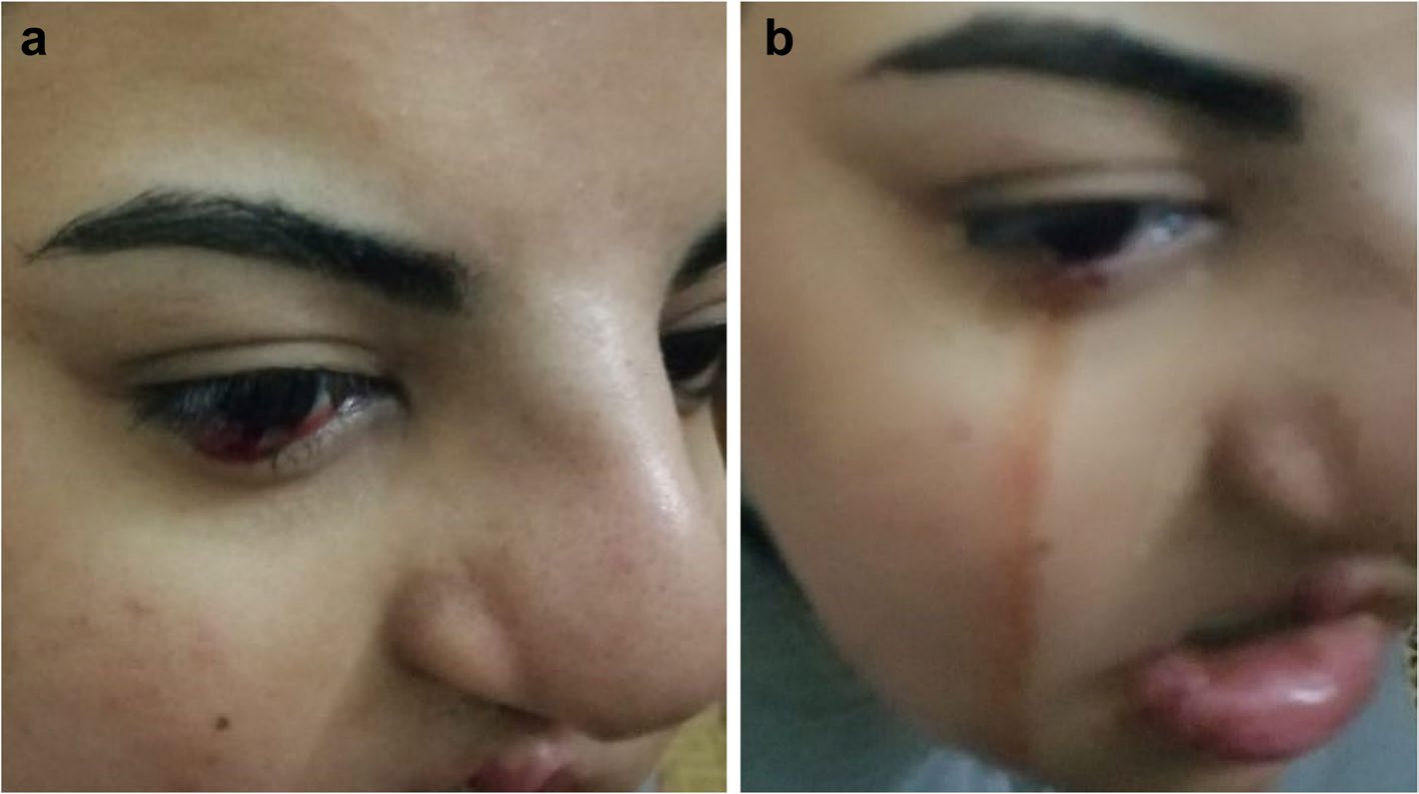
**a** and **b** Clinical picture showing the presence of the bloody tears in the eye

On the second day after the surgery, the patient reported overflow of tears mixed with bloody discharge (Fig. 1 a and b). It occurred independently, on drinking with a straw, and when applying any slight pressure on the cheeks. This symptom continued until the fourth postoperative day but decreasing in instances and intensity gradually, until complete disappearance. It began to cease after 2 days, disappearing completely on the fourth postoperative day.

**Fig. 2:**
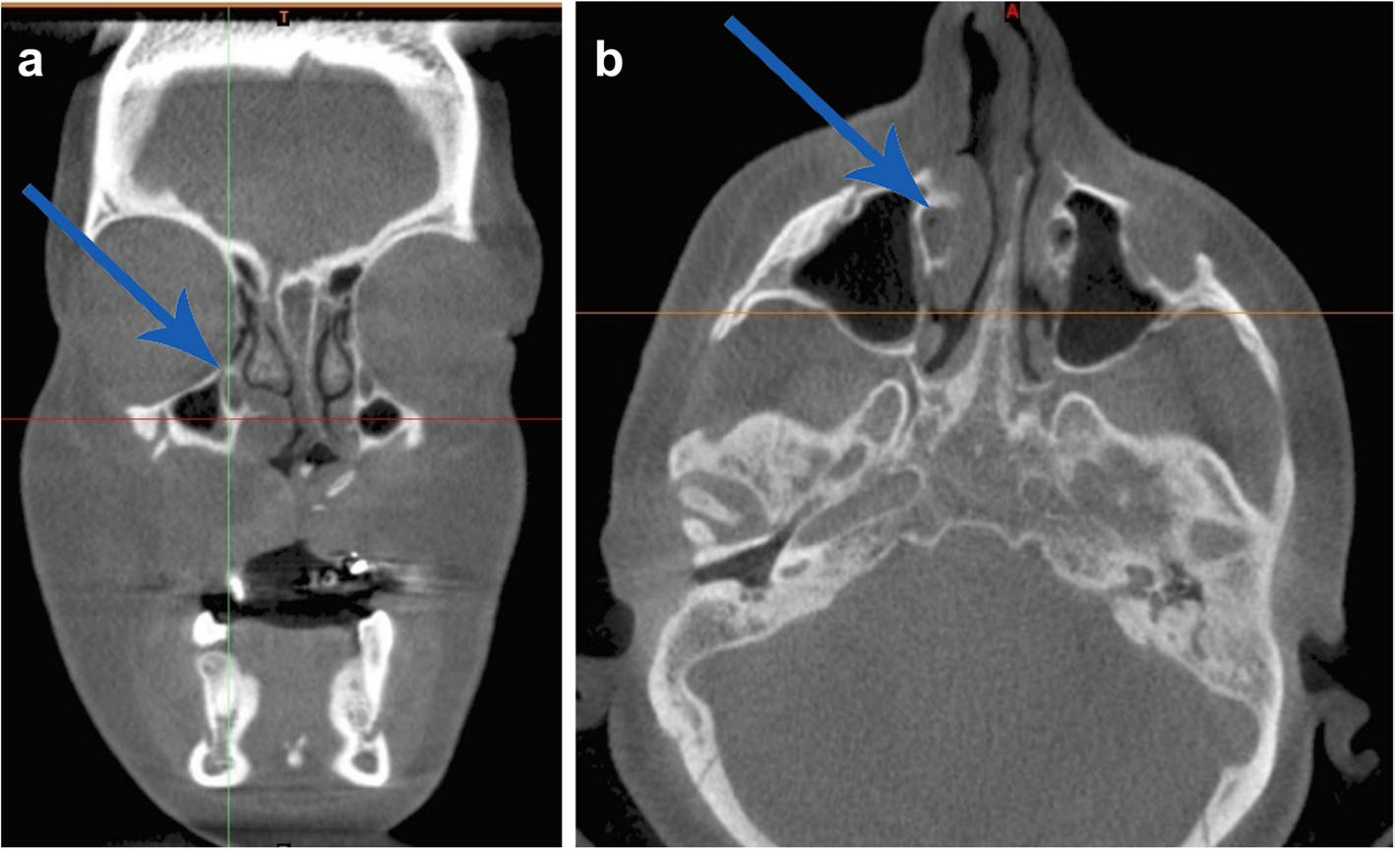
Coronal (**a**) and axial (**b**) tomograms indicating partial blockage of nasolacrimal ducts (indicated by arrows)

The CBCT scan of the patient was reassessed, as to search for a cause of this symptom, and the nasolacrimal duct was found to be partially blocked, showing an air-fluid level (Fig. 2 a and b).

## Discussion

Complications of LF-1 osteotomy are usually minor and transient, but not life threatening. They are related to many aspects, including injury to nerves, blood vessels, and/or bony canals [11]. Lanigan [13] states that “potential ophthalmic complications following Le Fort I osteotomies fall into four main categories: 1) a decrease in visual acuity, 2) extraocular muscle dysfunction, 3) neuroparalytic keratitis, and 4) lacrimal apparatus problems, including both epiphora and keratitis sicca.” Few have reported hemolacria as a complication of orthognathic surgery. This is typically encountered with other conditions such as fibromas, hemangiomas, malignant melanomas, hereditary hemorrhagic telangiectasis, and inflammatory granulomas [11]. Oozing of blood is frequently encountered in orthognathic surgeries as epistaxis, but not as hemolacria [4, 14].

Bloody tears or hemolacria is exit of blood from the lacrimal puncta along with excessive tearing [11]. The blood found in this hemolacria is usually retrograde hemorrhage due to disruption in the bony framework of the nasolacrimal canal due to maxillectomies and/or maxillary osteotomies or secondary to edema [4].

In the case described, the patient experienced hemolacria for 4 days after the operation, either spontaneously on drinking or due to pressure on the cheeks. The operation included a LF-1 osteotomy, which probably lead to a temporary obstruction of the nasolacrimal canal due to trauma, leading to this retrograde hemorrhage. Added to that is the soft tissue edema occurring after any surgical trauma. This created a blockage of the nasolacrimal canal preventing the outflow of the tears into the lacrimal puncta, thus causing the epiphora mixed with the blood from the retrograde hemorrhage. It is the authors’ belief that these factors had a greater effect on this case than most other cases undergoing LF-1 osteotomy due to two factors. First, the patient had a CLP defect, with underdevelopment of the nasal bones. This deprived the nasal soft tissues from the protection of the bony framework usually present. Second, the patient already had a congenital partial blockage of the nasolacrimal canal, which may be an anatomic variant associated with CLP.

Chrcanovic [11] reported a similar case of hemolacria, with discovery of blood in the eyes during the anesthesia recovery. Computed tomography scans revealed a fixation screw penetrating the nasolacrimal canal. A lacrimal duct endoscopy confirmed the source of bleeding around the penetrating screw. Hemolacria ceased after the removal of the screw. Humber [4] reported two cases that presented with both epistaxis and hemolacria occurring few days postoperatively. One case had greater manipulation of the bones than the usual due to difficult downfracture of the maxilla, owing to the higher density of the bone. The author also suggested that the cause was injury to small vessels, causing minor hemorrhage, with a path of least resistance into the nasolacrimal system. The other case was “more brisk and recurrent,” suggesting that a greater vessel has been traumatized, such as the sphenopalatine artery or one of its larger branches. Madattigowda [12] also reported a similar complication after LF-1 osteotomy.

Hemolacria has been described as a complication of other maxillofacial surgeries in only case in literature. It ensued after the placement of basal bone implants in an atrophic maxilla. The possible cause was a probable penetration of one of the implants into the nasolacrimal apparatus [15].

## Conclusion

Hemolacria is an infrequent finding after maxillofacial surgeries and may be associated with CLP surgeries more than other surgeries. In this case, it was easily managed, and surgeons should be more aware of it to try to prevent its occurrence, through careful management of tissues in the area of the nasolacrimal duct to prevent its injury.

## Data Availability

Not applicable.

## Abbreviations

NDO: Nasolacrimal duct obstruction
PANDO: Primary acquired nasolacrimal duct obstruction
SANDO: Secondary acquired nasolacrimal duct obstruction
LF-1: Le Fort I
CLP: Cleft lip and palate
BSSO: Bilateral sagittal split osteotomy
CBCT: Cone-beam computed tomography

## References

[1] Brodie SE, Fillit HM, Rockwood K, Woodhouse K (2010). Chapter 96 - aging and disorders of the eye. Brocklehurst’s textbook of geriatric medicine and gerontology.

[2] Paulsen F (2003). The human nasolacrimal ducts.

[3] Anatomy, head and neck, eye nasolacrimal. StatPearls Publishing 2022 29489208

[4] Humber CC, Lanigan DT, Hohn FI (2011). Retrograde hemorrhage (hemolacria) from the lacrimal puncta after a Le fort I osteotomy: a report of 2 cases and a review of the literature. J Oral Maxillofac Surg.

[5] Bartley GB (1992). Acquired lacrimal drainage obstruction: an etiologic classification system, case reports, and a review of the literature. Part 1. Ophthalmic Plast Reconstr Surg.

[6] Zapala J, Bartkowski AM, Bartkowski SB (1992). Lacrimal drainage system obstruction: management and results obtained in 70 patients. J Craniomaxillofac Surg.

[7] Mansukhani S. Congenital nasolacrimal duct obstruction. [Internet] Pediatric Oncall. Available through https://www.pediatriconcall.com/articles/pediatric-ophthalmology/congenital-nasolacrimal-duct-obstruction/congenital-nasolacrimal-duct-obstruction-introduction. Accessed June 2022.

[8] Ali MJ, Ali MJ (2018). Syndromic and systemic associations of congenital lacrimal drainage anomalies. Atlas of lacrimal drainage disorders.

[9] Worak S, Bengzon A (2018) Nasolacrimal duct obstruction and epiphora. Medscape [Available from: https://emedicine.medscape.com/article/1210141-overview

[10] Linberg JV, McCormick SA (1986). Primary acquired nasolacrimal duct obstruction. A clinicopathologic report and biopsy technique. Ophthalmology.

[11] Chrcanovic BR, Nunes FCF, Freire-Maia B (2013). Bloody tears after miniplate osteosynthesis for Le fort I osteotomy. J Oral Maxillofac Surg Med Pathol.

[12] Madattigowda R, Heliotis M (2017). Haemolacria a rare complication of Lefort I surgery. Br J Oral Maxillofac Surg.

[13] Lanigan DT, Romanchuk K, Olson CK (1993). Ophthalmic complications associated with orthognathic surgery. J Oral Maxillofac Surg.

[14] Girard A, Lopez CD, Chen J, Perrault D, Desai N, Bruckman KC (2021). Epistaxis after orthognathic surgery: literature review and three case studies. Craniomaxillofac Trauma Reconstr.

[15] Sabnis R, Lokare S, Rao S, Thakur D, Patel M (2019). Hemolacria and epistaxis as a complication of basal bone implant. J Dental Implants.

